# Combined expression of *JHDM1D/KDM7A* gene and long non-coding RNA *RP11-363E7.4* as a biomarker for urothelial cancer prognosis

**DOI:** 10.1590/1678-4685-GMB-2023-0265

**Published:** 2024-08-09

**Authors:** Glenda Nicioli da Silva, Isadora Oliveira Ansaloni Pereira, Ana Paula Braga Lima, Tamires Cunha Almeida, André Luiz Ventura Sávio, Renato Prado Costa, Kátia Ramos Moreira Leite, Daisy Maria Fávero Salvadori

**Affiliations:** 1 Universidade Federal de Ouro Preto, Escola de Farmácia, Departamento de Análises Clínicas, Ouro Preto, MG, Brazil.; 2 Instituto Butantan, Laboratório de Dor e Sinalização, São Paulo, SP, Brazil.; 3 Faculdade Centro Oeste Paulista, Departamento de Odontologia, Piratininga, SP, Brazil.; 4 Universidade do Oeste Paulista, Departamento de Ciências Médicas, Jaú, SP, Brazil.; 5 Hospital Amaral Carvalho, Jaú, SP, Brazil.; 6 Universidade de São Paulo, Faculdade de Medicina, Departamento de Cirurgia, São Paulo, SP, Brazil.; 7 Universidade Estadual Paulista, Faculdade de Medicina, Departamento de Patologia, Botucatu, SP, Brazil.

**Keywords:** Biomarker-oriented therapy, cancer progression, tumor biomarker, gene/lncRNA differential expression, urothelial carcinoma

## Abstract

Bladder cancer is the tenth most frequently diagnosed cancer globally. Classification of high- or low-grade tumors is based on cytological differentiation and is an important prognostic factor. LncRNAs regulate gene expression and play critical roles in the occurrence and development of cancer, however, there are few reports on their diagnostic value and co-expression levels with genes, which may be useful as specific biomarkers for prognosis and therapy in bladder cancer. Thus, we performed a marker lesion study to investigate whether gene/lncRNA expression in urothelial carcinoma tissues may be useful in differentiating low-grade and high-grade tumors. RT-qPCR was used to evaluate the expression of the *JHDM1D* gene and the lncRNAs *CTD-2132N18.2, SBF2-AS1, RP11-977B10.2*, *CTD-2510F5.4*, and *RP11-363E7.4* in 20 histologically diagnosed high-grade and 10 low-grade tumors. A protein-to-protein interaction network between genes associated with *JHDM1D* gene was constructed using STRING website. The results showed a moderate (positive) correlation between *CTD-2510F5.4* and *CTD2132N18.2*. ROC curve analyses showed that combined *JHDM1D* and *RP11-363E7.4* predicted tumor grade with an AUC of 0.826, showing excellent accuracy. In conclusion, the results indicated that the combined expression of *JHDM1D* and *RP11-363E7.4* may be a prognostic biomarker and a promising target for urothelial tumor therapy.

## Introduction

Bladder cancer is the tenth most frequently diagnosed cancer globally, and the most common occurring cancer of the urological system. In 2020, approximately 0.573 million new cases and 0.213 million deaths were estimated to occur worldwide due to bladder cancer (Sung et al., 2021). Up to 90-95% of urothelial carcinomas are identified as bladder cancers. At the time of diagnosis, bladder cancers can be classified as non-muscle-invasive (75%) or muscle-invasive (25%). Classification of high- or low-grade tumors is based on cytological atypia and cellular architecture and is an important prognostic factor (Martinez Rodriguez et al., 2017; Sanli et al., 2017; Lenis et al., 2020). Low-grade tumors are usually non-invasive, growing as superficial papillary protrusions restricted to the urothelium and lamina propria, with a high risk of recurrence (Martinez Rodriguez *et al*., 2017; Lenis *et al*., 2020). On the other hand, high-grade tumors may become muscle-invasive and progress to metastatic disease. A small percentage of low-grade tumors (10-15%) can progress to high-grade tumors and become invasive (Sanli *et al*., 2017).

Despite advancements in technology, cystoscopy, and urine cytology remain the gold standard for diagnosing and monitoring bladder cancer. The use of molecular tests may support the earlier detection of disease, risk stratification of patients, improved prediction of oncological outcomes, and optimization of target therapies. However, international guidelines have not yet incorporated new molecular assessments into daily clinical practice due to difficulty in identifying the appropriate scenario of use, as well as the lack of high-quality prospective trials, resulting in a low level of evidence (Giordano and Soria, 2020). In contrast, significant progress has already been made in terms of biomarker-oriented therapy, and newly identified biomarkers have been demonstrated to be essential in providing clinicians with the information needed to significantly expand their therapeutic arsenal (Jones et al., 2016; Scholtes et al., 2021).

Long noncoding RNAs (lncRNAs), defined as 500+ nucleotides-long non-protein-coding RNAs, have been increasingly shown to regulate gene expression at the epigenetic, transcriptional, and translational levels. Many lncRNAs are often found abnormally expressed in cancer and play critical roles in cancer occurrence and progression, acting as oncogenes as well as tumor suppressors (Mattick et al., 2023). Furthermore, tissue-specific lncRNAs with an ageing-associated expression pattern were observed in human tissues. Thus, lncRNA expression may reflect the tissue-specific fine-tuning of the ageing-associated process. However, no lncRNA has been associated with aging in bladder tissue (Marttila et al., 2020).

LncRNAs *SBF2-AS1* (Fu and Liu, 2021; Zha et al., 2021; Zhang et al., 2021), *RP11-977B10.2* (Liu *et al*., 2019), *CTD-2510F5.4*, and *RP11-363E7.4* (Wang and Qin, 2019; Chen et al., 2020) are shown to be implicated in carcinogenesis, as well as the histone demethylase *JHDM1D/KDM7A* (Osawa et al., 2011; Meng et al., 2020). However, there are few reports on their diagnostic value and co-expression levels, which may be useful as biomarkers for prognosis and therapy. Thus, we performed a marker lesion study to investigate whether gene/lncRNA expression in urothelial carcinoma tissues may be useful in differentiating between low- and high-grade tumors.

## Subjects and Methods

### Patients

Inclusion criteria for patient enrollment in the study were male patients, regardless of age, diagnosed with primary bladder tumors, who attended the University of Sao Paulo Biorepository (São Paulo, Brazil) and Amaral Carvalho Hospital (Jaú, São Paulo, Brazil). A total of 30 fresh bladder cancer tissue samples were collected by consecutive sampling. From those, 20 were histologically diagnosed as high-grade tumors and 10 as low-grade tumors (Table 1). All tumor samples were collected via transurethral resection and were histopathologically classified by a pathologist (K.R.M.L). The grading and stage were determined according to the World Health Organization (WHO) systems and Tumor-Node-Metastasis (TNM) 2017.

**Table 1 -:** Tumor grade and staging of the tumor tissue samples and patients’ ages.

Sample Number	Tumor Grade	Staging	Age
117	High Grade	2	69
113	High Grade	2	77
102	High Grade	2	72
110	High Grade	1	82
130	High Grade	2	46
225	High Grade	2	69
114	High Grade	1	70
104	High Grade	2	63
116	High Grade	1	53
128	High Grade	2	62
125	High Grade	1	70
123	High Grade	1	61
142	High Grade	2	76
169	High Grade	2	56
231	High Grade	2	61
167	High Grade	pTa	73
229	High Grade		54
248	High Grade	3	64
242	High Grade	3	75
163	High Grade	2	68
133	Low Grade	non-invasive	74
124	Low Grade	non-invasive	59
105	Low Grade	non-invasive	70
120	Low Grade	pTa	52
112	Low Grade		67
249	Low Grade	non-invasive	79
247	Low Grade	2a	91
145	Low Grade	pTa	72
151	Low Grade		89
147	Low Grade	pTa	78

pTA: Pappilary non-invasive bladder cancer.

The study was approved by the Ethics Committee of the Sao Paulo State University (protocol 48193715.6.0000.5411), and all methods were performed in accordance with the approved guidelines.

### Expression analysis 

Tissue biopsies were snap-frozen and stored at -80 °C. Total RNA was isolated using the RNeasy Mini Kit^®^ (Qiagen, Hilden, Germany) according to the manufacturer’s protocol. RNA concentration and purity were determined using a NanoDrop spectrophotometer (Thermo Scientific, Waltham, Massachusetts, EUA). RNA quality was analyzed using a 2100 Bioanalyzer (Agilent, Santa Clara, California, USA), and only samples with an RNA integrity number (RIN) ≥ 6.0 were used. Complementary DNA (cDNA) was synthesized using the High Capacity Kit (Applied Biosystems, Waltham, Massachusetts, USA) with random priming according to the manufacturer’s instructions. Expression levels of the *JHDM1D/KDM7A* gene (Pereira et al., 2023) and *CTD-2132N18.2, SBF2-AS1, RP11-977B10.2*, *CTD-2510F5.4* and *RP11-363E7.4* lncRNAs were analyzed using RT-qPCR. Endogenous reference genes (*HSPCB* and *ACTB*) were selected using the NormFinder software (Andersen et al., 2004).

Each PCR reaction was performed at a final volume of 10 µL, with the reaction mixture including 0.2 µg of the cDNA product, 0.75 µM of each specific primer, Fast Start DNA polymerase, reaction buffer, dNTPs, and SYBR green (Applied Biosystems). The forward and reverse primer sequences are listed in Table 2.

**Table 2-:** RT-qPCR primers used in this study.

Gene/lncRNA	Primer	Sequence	Reference
*CTD-2132N18.2*	CTD-2132N18.2 F	5’-GGCGTCAAGGTGGAGTTAGA-3’	Xia et al., 2021
	CTD-2132N18.2 R	5’-ATCCTCCTTTGCCATGCAGT-3’	
*CTD-2510F5.4*	CTD-2510F5.4 F	5’-GGTCTCTTGCTCTGTCACCC-3’	Wang and Qin, 2019
	CTD-2510F5.4 R	5’-GCACACCTGTAGTCCCAGTT-3’	
*RP11-363E7.4*	RP11-363E7.4 F	5’-CGACCACCTATTCCACTT-3’	Wang et al., 2018
	RP11-363E7.4 R	5’-GCCAGGAAGGCTCAAATC-3’	
*RP11-977B10.2*	RP11-977B10.2-F	5’-GGTCTTGAGTGGGGCAATCAGC-3’	Liu et al., 2019
	RP11-977B10.2-R	5’-GAGGTCTTTGCAGGAGCCGATG-3’	
*HSPCB*	HSPCB F	5’-AAGAGAGCAAGGCAAAGTTTGAG-3’	Andersen et al., 2004
	HSPCB R	5’-TGGTCACAATGCAGCAAGGT-3’	
*ACTB*	ACTB-S	5’-AGAAGGAGATCACTGCCCTGGCACC-3’	Kondo et al., 2017
	ACTB-A	5’-CCTGCTTGCTGATCCACATCTGCTG-3’	
*JHDM1D*	JHDM1D-S	5’-TATTCAGGGCATGCTGTCTATG-3’	Kondo et al., 2017
	JHDM1D-AS	5’-GGGATCCTGGAGAGAGTTTCTT-3’	
*SBF2-AS1*	SBF2AS1S	5’-CACGACCCAGAAGGAGTCTAC-3’	Chen et al., 2019
	SBF2AS1AS	5’-CCCGGTACCTTCCTGTCATA-3’	

The thermal cycling conditions for all genes comprised a temperature profile at 95 °C for 10 min, for initial denaturation, followed by 40 cycles at 95 °C for 15 s and 60 °C for 60 min. RT-qPCR specificity was assessed through melting curves analyses of the amplification products, and the reaction efficiency was estimated using the slope of the standard curve. Standard curves were designated based on the Ct values of cDNA serial dilutions. The relative expression of gene transcripts was calculated using ∆Ct and 2^(-∆Ct)^formulas concerning multiple genes (Vandesompele et al., 2002).

“The Atlas of ncRNAs in cancer” (TANRIC - https://www.tanric.org) database, which comprises data from expression profiles of lncRNAs and patient survival outcomes, was used to access the survival analysis of groups with high or low expression of the investigated lncRNAs (Li et al., 2015). Survival analyses were accessed by Cox Regression analysis, and the significant differences between the survival curves of the two groups were analyzed by Log-Rank P-value. The R2: Genomics Analysis and Visualization Platform (https://hgserver1.amc.nl/cgi-bin/r2/main.cgi), was used to access the Cox Regression analysis for *JHDM1D/KDM7A* gene.

The STRING 11.5 website (https://string-db.org/) and MCL clustering algorithms were used to create a protein-to-protein interaction network (PPI) between genes associated with *JHDM1D* gene.

### Statistical analysis

The nonparametric Mann-Whitney test was used for differential gene expression analyses, and the values are expressed as the mean ± SD. The correlation between the differentiated values was examined using Spearman’s rank correlation test (r = correlation coefficient), according to Akoglu (2018). A receiver operating characteristic (ROC) curve was constructed, and the area under the curve (AUC) was calculated to assess the specificity and sensitivity of the predicted gene/lncRNA in differentiating high- and low-grade tumors. Statistical significance was set at *p* < 0.05. Data analysis was performed using GraphPad Prism version 6 and IBM SPSS 17.0.

## Results

**Figure 1 -:**
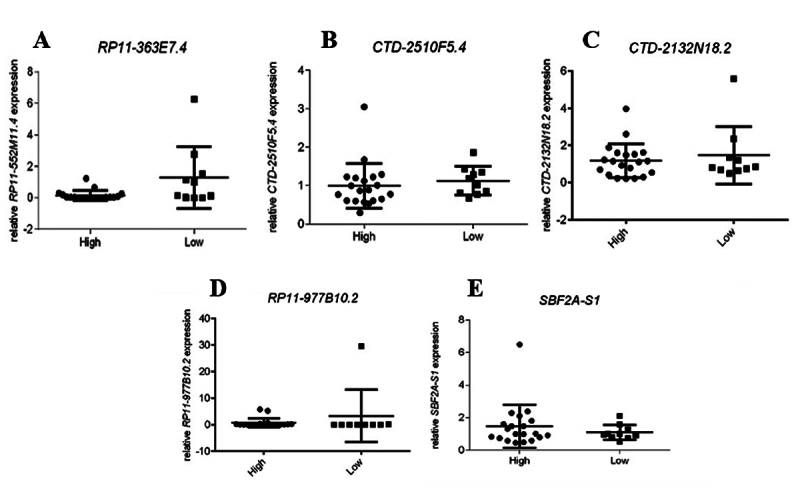
Relative expression levels of lncRNAs *RP11-363E7.4* (A), *CTD-2510F5.4* (B)*, CTD-2132N18.2* (C)*, RP11-977B10.2* (D)*,* and *SBF2-AS1* (E) in patients with low- and high-grade bladder tumors. Values are expressed as the mean ± SD. *p < 0.05.

No significant differential expression of the lncRNAs *CTD-2132N18.2, SBF2-AS1, RP11-977B10.2*, *CTD-2510F5.4*, and *RP11-363E7.4* was detected between low- and high-grade tumors (Figure 1). Although not statistically significant, lower expression of *RP11-363E7.4* (*p* = 0.1041) was observed in high-grade tumors compared to low-grade tumors.

**Figure 2 -:**
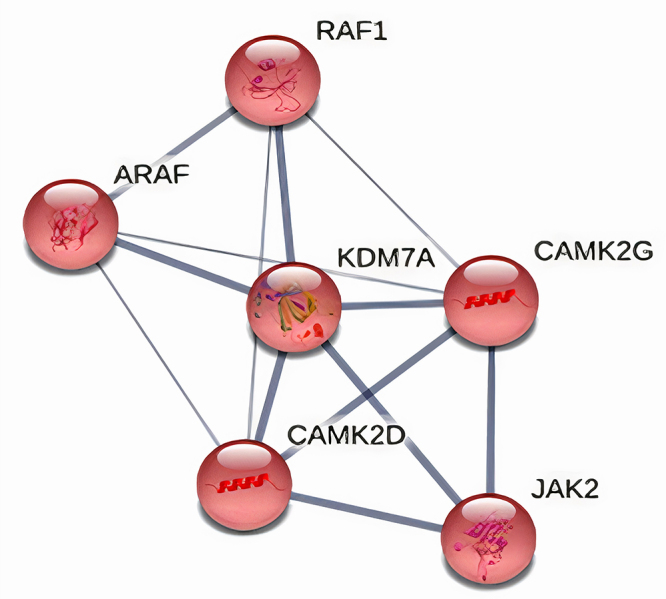
*JHDM1D/KDM7A* gene interaction network, created using String 11.5 software and MCL clustering algorithms. Line thickness indicates data support strength

As formerly presented by Pereira et al (2023), *JHDM1D/KDM7A*gene expression was 2.01 times greater in high-grade tumors than in low-grade tumors. Figure 2 shows the *JHDM1D/KDM7A* interaction network. The analysis highlights the relationship between *JHDM1D/KDM7A* and *RAF1*, *JAK2, ARAF*, and *CAMK2*.

**Figure 3 -:**
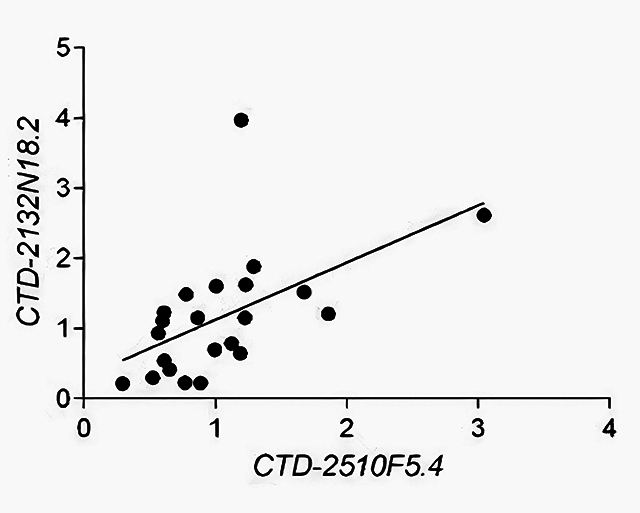
Correlation analysis showing a moderate (positive) correlation between the lncRNAs *CTD-2132N18.2* and *CTD-2510F5.4* expression in both low- and high-grade tumor samples. Spearman correlation analysis. r = 0.6488, *p* < 0.0011.

A correlation analysis was performed to investigate the relationship between *JHDM1D/KDM7A* and the lncRNAs *CTD-2132N18.2, RP11-977B10.2, CTD-2510F5.4, SBF2-AS1,* and *RP11-363E7.4* in both low- and high-grade tumors. This analysis using low- and high-grade samples revealed a moderate (positive) correlation between *CTD-2510F5.4* and *CTD-2132N18.2* expression (r = 0.6488, *p* < 0.0011) (Figure 3). 

**Figure 4 -:**
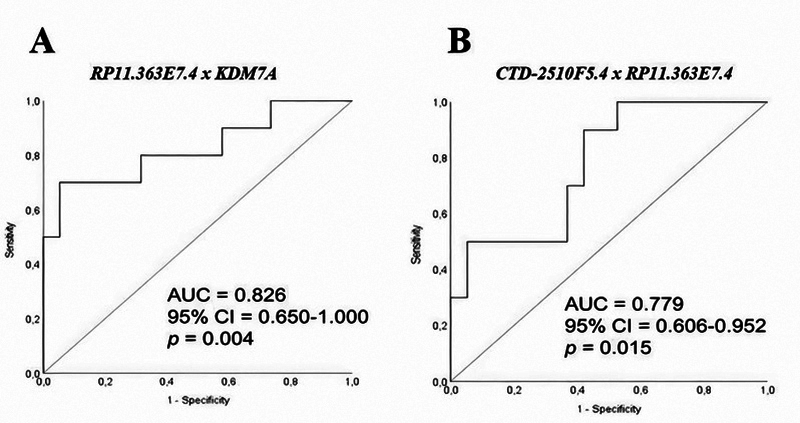
Receiver operating characteristics (ROC) curves using the combined expression of gene/lncRNA KDM7A/*RP11-363E7.4* (A) and lncRNAs *CTD-2510F5.4*/ *RP11-363E7.4* (B) to access accuracy to distinguish between low- and high-grade tumors.

The ability of each isolated lncRNA and the interactions between gene/lncRNA and lncRNA/lncRNA to predict the different tumor subgroups was also tested. Receiver operating characteristics (ROC) curve analyses showed that combined *JHDM1D/KDM7A* gene and *RP11-363E7.4* lncRNA predicted tumor grade with an AUC of 0.826 (*p* = 0.004), showing excellent discrimination capacity (Mandrekar, 2010). Combined expression of *CTD-2510F5.4* and *RP11-363E7.4* also showed an acceptable potential diagnostic value, with an AUC of 0.779 (*p* = 0.015), demonstrating their ability to distinguish between low- and high-grade tumors (Figure 4) (Mandrekar, 2010).

## Discussion

Several studies have revealed that lncRNA show highly specific expression patterns in different biological contexts, and their abnormal expression is associated with the progression and prognosis of human malignancies (Fu and Liu, 2021; Gao et al., 2021; Pereira et al., 2023). Thus, a better understanding of the interaction between genes and lncRNAs may be useful in cancer diagnosis, prognosis, and treatment. Based on this rationale, we conducted a marker lesion study using gene/lncRNA expression to distinguish, with high specificity, between low-and high-grade bladder tumors.

The histone demethylase *JHDM1D* gene (also known as *KDM7A*) is a member of the plant homeodomain (PHD) finger protein (PHF) family of PHD and JmjC domain-containing histone demethylases and participates in epigenetic regulation (Klose et al., 2006). This gene also regulates many biological processes, including differentiation, development, and growth of several cancer cells (Yang et al., 2019; Meng et al., 2020). In bladder cancer cell lines, *JHDM1D* knockdown led to impaired cell growth, increased cell death, and reduced rates of cell migration (Lee et al., 2018). Indeed, a study from Pereira et al. (2023) showed different expression levels of *JHDM1D* in low- and high-grade tumors, suggesting a possible role of this gene in bladder tumor progression. In addition, we found that the interaction network involving *JHDM1D/KDM7A* was associated with *RAF1*, *ARAF*, *JAK2,* and *CAMK2* activation, reinforcing the involvement of *JHDM1D/KDM7A* in tumor aggressiveness and progression. As per the literature, these four genes also play a role in carcinogenesis. *RAF* kinases normally function as activators of the mitogen-activated protein kinase (MAPK) signaling pathway, which indirectly regulates cell proliferation and survival (Montagut and Settleman, 2009). *ARAF,* also required for MAPK activation in a variety of cancer types (e.g., colorectal, pancreatic, and breast cancers), is associated with the migration and invasiveness of tumor cells (Mooz et al., 2014). The exact role of *JAK2* signaling in solid cancers is unclear, but *JAK2* inhibition may prevent disease progression through restriction of malignant cell phenotypes (Harry et al., 2012). The emerging role of the *CAMK2* gene in the regulation of cancer progression, especially proliferation, cell cycle, and metastasis, and in therapy response has also been reported (Wang et al., 2015) Therefore, *JHDM1D* gene expression levels were chosen to be correlated with lncRNAs expression to access our prognostic marker lesion study.

Combined gene/lncRNA or lncRNA/lncRNA expression can be used to achieve more accurate diagnosis and prognosis when compared to the analysis of the gene/lncRNA alone. Indeed, Pereira et al. (2023) found a moderate positive correlation between *JHDM1D* gene and lncRNA *JHDM1D-AS1* (an antisense transcript from *JHDM1D*) expression in high-grade tumors. In addition, the combination of*JHDM1D*and*JHDM1D*-AS1 showed potential prognostic value in distinguishing between low- and high-grade bladder tumors. In our study, no correlation was found between *JHDM1D* gene and the analyzed lncRNAs. Nevertheless, although a positive correlation between the lncRNAs *CTD-2132N18.2* and *CTD-2510F5.4* was observed, no diagnostic predictive value was detected. Similarly, *SBF2-AS1* and *RP11-977B10.2* levels also failed to differentiate between low- and high-grade tumors. Therefore, the lncRNAs *CTD-2132N18.2, CTD-2510F5.4, SBF2-AS1,* and *RP11-977B10.2* were not associated with bladder tumor progression.

The best potential diagnostic values of combined detection occurred with lncRNA *RP11-363E7.4,* a recently discovered novel lncRNA. *JHDM1D/RP11-363E7.4* combination appeared to have excellent diagnostic value, as predicted by the ROC curve. Furthermore, the combination of lncRNAs *CTD-2510F5.4* and *RP11-363E7.4* also showed an acceptable potential diagnostic value. Despite the lack of statistical significance (*p* = 0.1041), decreased expression of *RP11-363E7.4* in high-grade tumors compared to low-grade tumors may have some clinical significance. This finding suggests that downregulation of this lncRNA is associated with tumor aggressiveness. Similarly, *RP11-363E7.4* downregulation was observed in gastric cancer, with its higher expression correlated with better overall survival in cancer patients (Wang et al., 2018). Although the functional role and molecular mechanisms are still unclear, a recent study by (Chen et al., 2020) showed that *RP11-363E7.4* can function as a tumor suppressor by inhibiting proliferation, migration, and invasion, and inducing apoptosis.

The prognostic biomarkers proposed aimed at predicting the tumor grade, however, the lncRNA and/or gene expression levels may also be valuable when evaluating clinical patient outcomes. Although our study has not revealed a significant difference in the expression of the mentioned lncRNAs between high and low-grade bladder tumors, the Log-Rank Test applied to Cox regression analysis showed that higher expression of lncRNAs *CTD-2132N18.2* (Log-Rank P-value = 0.04908) and *RP11-977B10.2* (Log-Rank P-value = 0.0060563) were associated with lower survival probability when compared to the group with lower expression of these lncRNAs. No significant differences in the outcome were found between groups with lower and higher expression of lncRNAs *SBF2-AS1* and *RP11-363E7.4*, and no data about lncRNA *CTD-2510F5.4* was found in TANRIC database (Li et al., 2015). Moreover, a significantly lower survival probability of groups with high expression of *JHDM1D/KDM7A* gene, which showed potential prognostic value in predicting tumor grade in our study, was also observed (Cox p-value = 0.00217). 

It is important to highlight that the number of tissue samples analyzed may have been a limitation of the current study. A greater number of bladder tumor specimens may be able to more clearly demonstrate the link between certain gene/lncRNA combinations and tumor progression. LncRNA *RP11-363E7.4* and *JHDM1D* silencing in low- and high-grade cell lines, and subsequently resulting changes in biological behavior should also be considered.

In conclusion, this study revealed that the combined expression of *JHDM1D/RP11-363E7.4* may predict tumor progression, and this combination may serve as an attractive prognostic biomarker and a promising target for urothelial carcinoma treatment.
